# Solitary Parotid Metastasis as an Atypical Site of Recurrence in Lung Squamous Cell Carcinoma: A Diagnostic Difficulty

**DOI:** 10.7759/cureus.108111

**Published:** 2026-05-01

**Authors:** Mamadou Alpha Prateaux, Mohamed Amine Haouane, Issam Rharrassi, Mohamed Amine Azami

**Affiliations:** 1 Department of Pathology, Faculty of Medicine and Pharmacy, Cady Ayyad University / Avicenna Military Hospital, Marrakech, MAR; 2 Department of Pathology, Cady Ayyad University / Avicenna Military Hospital, Marrakech, MAR

**Keywords:** lung squamous cell carcinoma, metachronous cancer, oncology, parotid gland metastasis, salivary gland neoplasm

## Abstract

Lung squamous cell carcinoma (LSCC) rarely metastasizes to the parotid gland, with only a handful of cases reported in the literature. Its clinical and pathological presentations closely mimic those of primary parotid squamous cell carcinoma (PSCC). Differentiating solitary metastasis from metachronous primary squamous cell carcinoma (SCC) in patients with a history of smoking and lung cancer presents a significant diagnostic challenge. A 55-year-old man with a history of heavy smoking presented with a solitary parotid mass two years after curative LSCC resection. Excisional biopsy with histological and immunohistochemical analyses confirmed SCC. Based on (1) a clear history of a primary tumor with a known metastatic propensity, (2) the absence of other primary sites on whole-body imaging, (3) histological features (highly consistent with the primary tumor type and no evidence of squamous metaplasia and dysplasia in the ductal or acinar epithelium), and (4) an immunohistochemical profile that effectively ruled out common primary parotid neoplasms, we retained solitary parotid metastasis as LSCC recurrence. However, definitive comparative molecular profiling, which is crucial for distinguishing recurrence from a second primary tumor, could not be performed because of resource limitations. Beyond the diagnostic challenge, this case highlights the complex intersection between rare metastatic sites and the risk of developing a secondary malignancy. In such scenarios, the decision to proceed with systemic therapy without definitive molecular proof of clonal relatedness depends on a robust convergence of clinical context, consistent imaging, and highly suggestive histopathological and immunohistochemical findings. It is imperative that traditional pathology and advanced molecular studies converge to provide not only a diagnosis but also a precise biological understanding of the disease.

## Introduction

Lung squamous cell carcinoma (LSCC) frequently metastasizes to the brain, bones, liver, and adrenal glands [1]. Metastasis to the parotid gland is rare and typically occurs sporadically, often alongside widespread disease at presentation [2].

We report the case of a 55-year-old man with a substantial history of smoking and a prior diagnosis of LSCC, who subsequently developed a solitary mass in the right parotid gland three years following surgical resection. An excisional biopsy and histological analyses confirmed the diagnosis of squamous cell carcinoma (SCC). Could this represent a recurrence of his prior LSCC or a metachronous primary parotid squamous cell carcinoma (PSCC)?

Distinguishing solitary metastases from metachronous primary SCC in individuals with a history of smoking and lung cancer represents a considerable diagnostic problem. This distinction is crucial, as a metachronous primary tumor may necessitate intensive local treatment, whereas a solitary metastasis can often be addressed through localized interventions, such as surgery or radiation, within the framework of systemic disease or may suggest the requirement for an adjustment in systemic therapy [3]. Nonetheless, in such scenarios, the decision to proceed with systemic therapy without definitive molecular proof of clonal relatedness is difficult. This case underscores the critical diagnostic process based on the robust convergence of clinical context, consistent imaging, and highly suggestive histopathological and immunohistochemical findings.

## Case presentation

A 55-year-old former smoker with a 35-pack-year history presented to the head and neck department for the evaluation of a right parotid mass. The patient’s oncologic history included stage IB (pT2aN0M0) LSCC of the right upper lobe, which was treated with lobectomy and adjuvant chemotherapy three years prior. He was in clinical remission, and annual surveillance computed tomography (CT) scans showed no evidence of disease. He reported a non-tender, gradually enlarging lump below his right ear for two months.

Physical examination revealed a 2 cm, firm, and mobile mass in the right parotid tail. Facial nerve function was intact, and cervical lymphadenopathy or cutaneous lesions were not observed. Ultrasonography confirmed the presence of a solid vascular parotid lesion. A subsequent CT scan revealed a solitary, well-defined, 1.7 cm enhancing mass confined to the superficial lobe of the right parotid gland (Figure 1A). Given the patient’s LSCC history and the indeterminate nature of the mass on imaging, a multidisciplinary team recommended whole-body positron emission tomography (PET) before excision. The PET scan revealed a fluorodeoxyglucose (FDG)-avid, well-circumscribed mass within the superficial lobe of the right parotid gland, measuring approximately 1.7 cm in axial diameter (Figure 1B). No additional tumors were identified.

**Figure 1:**
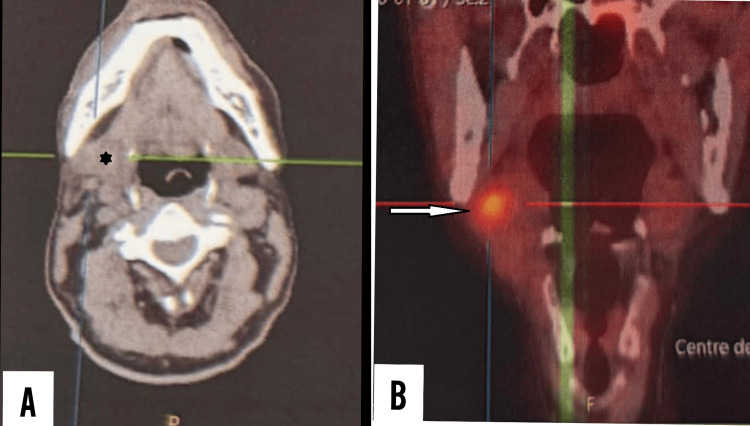
Imaging of the solitary parotid mass (A) Axial computed tomography (CT) scan illustrating a well-defined, enhancing, heterogeneous mass localized to the right parotid gland (star). (B) A coronal positron emission tomography (PET) scan reveals an FDG (fluorodeoxyglucose)-avid lesion within the right parotid gland, indicative of tumor metabolic activity (arrow).

After superficial parotidectomy, the surgical specimen was serially sectioned transversely at 5 mm intervals. Within the parenchyma, a well-circumscribed, firm nodule measuring 1.7 × 1 × 1 cm was identified. The cut section exhibited a solid, homogeneous appearance with a faintly lobulated contour surrounded by a thin rim of grossly normal yellow-tan parotid tissue (Figure 2A). Microscopic examination revealed nests and sheets of malignant epithelial cells with intercellular bridges, exhibiting well-defined but non-keratinized cytoplasm with large central pleomorphic nuclei and coarse chromatin, consistent with poorly differentiated SCC of the parotid gland (Figures 2B, 2C). No evidence of squamous metaplasia or dysplasia was observed in the ductal or acinar epithelium of the adjacent parenchyma, further supporting the diagnosis of metastatic SCC. Mitotic activity was low. There was no evidence of necrosis, perineural infiltration, or vascular or lymphatic invasion. The surgical margin was 4 mm.

**Figure 2:**
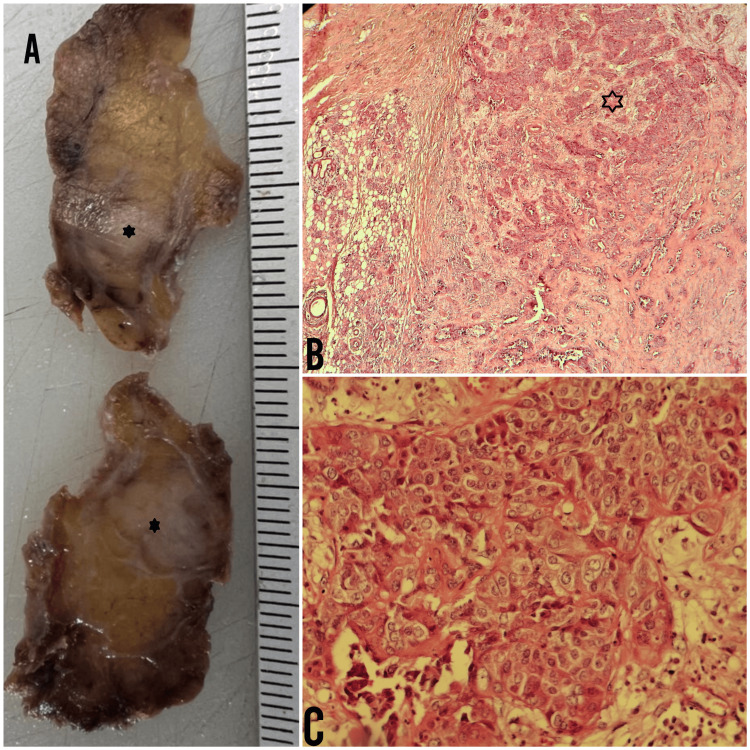
Macroscopic and microscopic characteristics of the PSCC (A) Gross view of the right parotid gland revealed a well-circumscribed, firm nodule with a white-tan color and a solid, homogeneous, faintly lobulated cut surface (star). (B) A low-power histological image of the parotid gland tumor illustrates a malignant epithelial neoplasm composed of nests and sheets of tumor cells within the parotid parenchyma (stars; H&E). (C) A high-power histological view showing poorly differentiated squamous cell carcinoma cells exhibiting intercellular bridges and a lack of keratinization (H&E). H&E: hematoxylin and eosin staining; PSCC: parotid squamous cell carcinoma

Immunohistochemically, the tumor cells' nuclei showed a strong positive reaction for p40 and a heterogeneous cytoplasmic reaction for cytokeratin (CK) 5/6 and CK7 (Figure 3). Additionally, other markers, including the androgen receptor (AR), smooth muscle actin (SMA), gross cystic disease fluid protein-15 (GCFDP-15), S100 protein, cluster of differentiation (CD) 117, and CK20, were negative, thereby excluding the possibility of other parotid carcinomas (ductal, mucoepidermoid, epithelial myoepithelial, and adenoid cystic), supporting the diagnosis of SCC.

**Figure 3:**
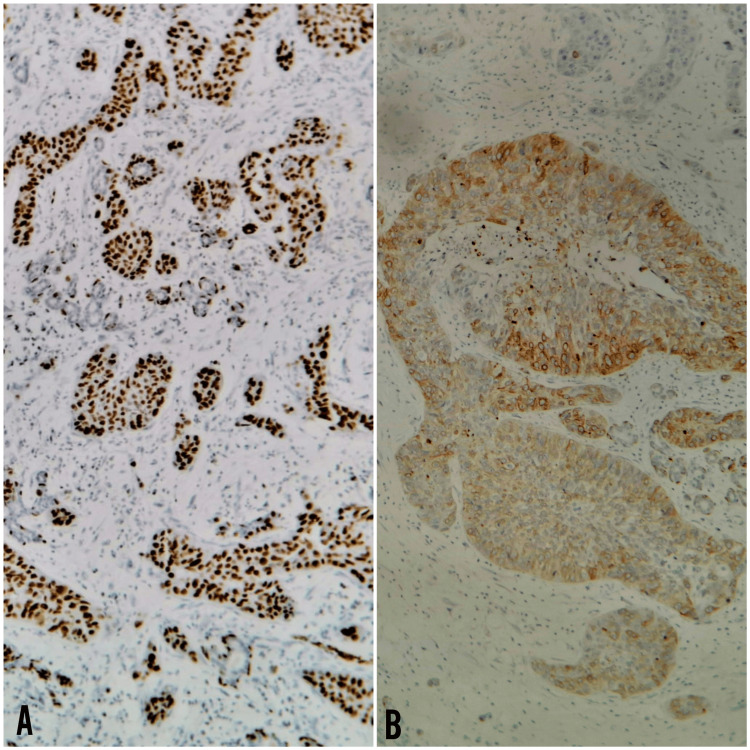
Immunostaining of PSCC Immunostaining of PSCC. The tumor cells showed a strong positive nuclear reaction for p40 (A) and a heterogeneous cytoplasmic reaction for CK5/6 (B). PSCC: parotid squamous cell carcinoma

Finally, based on (1) a clear history of a primary tumor with a known metastatic propensity; (2) the absence of other primary sites on whole-body imaging; (3) histological features (highly consistent with the primary tumor type and no evidence of squamous metaplasia or dysplasia in the ductal or acinar epithelium); and (4) an immunohistochemical profile that effectively ruled out common primary parotid neoplasms, solitary parotid metastasis as LSCC recurrence was strongly suggested, pending definitive comparative molecular studies. Systemic adjuvant therapy was recommended in a multidisciplinary meeting. The patient initiated systemic adjuvant chemotherapy with cisplatin and docetaxel and showed no evidence of disease progression at the 10-month follow-up.

## Discussion

Although most parotid masses are benign or malignant salivary tumors, they can also be sites of metastatic disease, primarily in the head and neck region [4]. However, metastasis from infraclavicular primary SCCs, especially LSCC, is rare and poses a significant diagnostic problem [5]. In fact, parotid gland lymph nodes can harbor metastases from both head and neck primaries and distant sites [6]. Histologically and immunohistochemically, PSCCs are indistinguishable from other SCCs because they may exhibit similar profiles (positivity for p40 and CK5/6) [7]. In this case, the diagnosis was made with a high degree of concordance across multiple modalities: the combination of (1) a clear history of a primary tumor with a known metastatic propensity; (2) the absence of other primary sites on whole-body imaging; (3) histological features (highly consistent with the primary tumor type and no evidence of squamous metaplasia and dysplasia in the ductal or acinar epithelium); and (4) an immunohistochemical profile that effectively ruled out common primary parotid neoplasms. This represents a compelling argument for metastasis [8]. Although this provided strong evidence, the ideal or gold-standard approach would involve molecular profiling, a method that is unfortunately unavailable in this patient’s case owing to resource limitations [9]. This leaves a degree of uncertainty regarding clonal relatedness, potentially impacting the most precise long-term management strategies, despite the current systemic therapy approach.

Molecular profiling is a valuable tool for distinguishing between metastatic and metachronous primary tumors. Comparative genetic studies that analyze the genetic fingerprints of tumors can offer more definitive insights. Metastasis represents clonal spread, indicating that the parotid tumor is a direct descendant of the original lung cancer clone [9,10]. Consequently, the two tumors share a set of identical tumor-specific genetic alterations (driver mutations, copy number variations, and chromosomal rearrangements) in the treated lung cancer cells. For example, multiple somatic mutations in genes such as *EGFR*, *ROS1*, *TP53*, *PIK3CA*, *CDKN2A*, and *NOTCH1*,* *each exhibiting the same nucleotide change and similar variant allele frequencies, may be observed. The presence of two or more unique shared truncal mutations is considered definitive evidence of clonal relatedness [10]. Conversely, a metachronous primary neoplasm represents a second independent carcinogenic event arising from the same carcinogenic field. Although both tumors may share common background field mutations, such as *TP53*, owing to tobacco exposure, their patterns of specific somatic driver alterations are fundamentally distinct [11].

This case illustrates a classic diagnostic challenge in oncology: differentiating between solitary metastases and metachronous second primary carcinomas in patients with a significant smoking history. This highlights the critical need for advanced molecular profiling and a deeper understanding of carcinogenesis to guide clinical decision-making [12]. The concept of field carcinogenesis suggests that chronic exposure to carcinogens, such as tobacco smoke, causes widespread genetic and molecular damage across the entire epithelial surface of the aerodigestive tract [13,14]. This, in turn, leads to premalignant changes, predisposing patients to the development of multiple independent primary tumors over time. Research indicates that patients treated for an initial head and neck or lung cancer face a lifelong, significantly elevated risk of developing a second primary malignancy in the lungs, head, neck, or esophagus, with annual risks ranging from 3% to 7% [15].

Classifying this lesion as a metastasis has profound implications, as it reclassifies the patient’s lung cancer from a presumed cured, localized state to a metastatic state, significantly altering the prognosis and treatment. This multi-pronged, converging evidence serves as a sufficient pragmatic threshold to initiate systemic adjuvant therapy, acknowledging the inherent uncertainty but prioritizing timely, potentially life-saving treatment. Although isolated metastases may be surgically excised, the focus must now be on systemic disease control (SDC). This typically involves systemic therapy, including immunotherapy, targeted therapy, and chemotherapy, guided by the tumor’s molecular profile, rather than aggressive local therapy, such as radical parotidectomy with neck dissection, which is the standard treatment for primary high-grade parotid carcinoma [16,17]. This underscores the need for lifelong vigilant surveillance in such patients, not only for the recurrence of primary lung cancer but also for the development of true second primaries in carcinogen-exposed fields [17].

## Conclusions

This case demonstrates more than just a rare metastatic site; it highlights a classic diagnostic challenge in oncology: differentiating between solitary oligometastasis and metachronous second primary SCC in patients with a significant smoking history. In patients with a history of smoking and SCCs, any new SCC should be presumed to be a metastasis or second primary malignancy after rigorous investigation with meticulous histopathological, immunohistochemical, and molecular studies. This distinction is critical, as it dictates contrasting management strategies: systemic therapy for metastatic disease versus curative-intent local therapy for new localized cancers. Finally, this case exemplifies the modern management of patients with a history of field carcinogenesis who develop new lesions. It advocates an integrated, multidisciplinary diagnostic approach that converges traditional pathology and advanced molecular studies. This approach not only provides a diagnosis but also a precise biological understanding of the disease, enabling potential personalized and effective treatment strategies.
